# On the Various Numerical Techniques for the Optimization of Bone Scaffold

**DOI:** 10.3390/ma16030974

**Published:** 2023-01-20

**Authors:** Jiongyi Wu, Youwei Zhang, Yongtao Lyu, Liangliang Cheng

**Affiliations:** 1 Department of Engineering Mechanics, Dalian University of Technology, No. 2 Linggong Road, Dalian 116024, China; 2 State Key Laboratory of Structural Analysis for Industrial Equipment, Dalian University of Technology, No. 2 Linggong Road, Dalian 116024, China; 3 Department of Orthopeadics, Affiliated Zhongshan Hospital of Dalian University, No. 6 Jiefang Street, Dalian 116001, China

**Keywords:** bone scaffolds, bio-porous structures, mechanical properties, numerical techniques, optimized design

## Abstract

As the application of bone scaffolds becomes more and more widespread, the requirements for the high performance of bone scaffolds are also increasing. The stiffness and porosity of porous structures can be adjusted as needed, making them good candidates for repairing damaged bone tissues. However, the development of porous bone structures is limited by traditional manufacturing methods. Today, the development of additive manufacturing technology has made it very convenient to manufacture bionic porous bone structures as needed. In the present paper, the current state-of-the-art optimization techniques for designing the scaffolds and the settings of different optimization methods are introduced. Additionally, various design methods for bone scaffolds are reviewed. Furthermore, the challenges in designing high performance bone scaffolds and the future developments of bone scaffolds are also presented.

## 1. Introduction

Bone defects are one of the major challenges in orthopedics, with approximately 2.2 million bone graft procedures performed worldwide each year and the annual cost of these procedures approaching $2.5 billion [1,2]. Tissue transplants have been used in humans for tissue repair since at least 1660 [3]. Allogeneic bone grafting is considered an effective option for bone repair, but the problem of allograft immune rejection seriously affects its use in clinical practice. However, the widely used artificial bone substitutes sometimes produce foreign body reactions due to the mismatch in biocompatibility and mechanical properties and the inability to participate in normal metabolic activities [4]. For this reason, new methods and techniques are explored to solve the challenges of bone defect treatment.

Tissue engineering is a new discipline that has emerged in recent years with the development of modern biomaterials technology and cellular biotechnology and other emerging technologies to develop biological substitutes for repairing and maintaining or promoting functional and morphological recovery of tissues or organs after injury. Tissue engineering research consists of four main elements: signaling molecules, target cells that respond to the regulation of the signaling molecules, scaffold materials, and recipient beds with good blood supplies [5]. Bone tissue scaffolds not only provide a three-dimensional environment for cell growth and metabolism but also play a supporting role. Thus, how to construct a scaffold that meets the requirements is one of the priorities of bone tissue scaffold research. The ideal bone scaffold should have properties such as bioactivity, biodegradability, biocompatibility, mechanical support, good porosity, and the ability to deliver material, and the scaffold gradually degrades as new bone tissue proliferates and grows until it completely replaces the scaffold and grows into new bone [6,7,8,9,10]. However, the traditional methods for producing bone tissue scaffolds, such as particle elution, electrostatic spinning, phase separation/freeze-drying, and gas forming, cannot precisely control the size of the scaffold pores, and the connectivity of the pores cannot be guaranteed, which cannot simulate the anisotropy of natural bone [11]. The structural shape also cannot match the anatomical morphology of the bone defect site, and the preparation of personalized bone tissue scaffolds cannot be realized [12,13].

Bone tissue engineering has benefited from the development of additive manufacturing technologies in the past few years. Scaffold geometric parameters such as porosity, pore size, shape, and interconnectivity can be precisely controlled by additive manufacturing techniques. Tissue scaffold structures can be fabricated to meet functional requirements. The additive manufacturing techniques that can produce tissue scaffolds include selective laser sintering (SLS), selective laser melting (SLM), electron beam melting (EBM), and binder jetting (BJ) [14].

With the increasing maturity of additive manufacturing technology and computer-aided design (CAD) technology, it has become possible to achieve the design and fabrication of complex controllable porous scaffolds. To better mimic the function of natural bone structures, various methods have been proposed for the design of bone tissue scaffolds. However, in the investigations of bone tissue scaffolds, most review articles focus on the design and manufacturing methods of tissue scaffolds, while only a few of them systematically introduce the optimal design methods of tissue scaffolds [15,16]. The main purpose of this paper is to review the existing computer-aided tissue scaffold optimization design methods and give the future development trends of tissue scaffolds in light of the current problems faced in the design of scaffolds.

## 2. Current Methods in the Optimization of Bone Scaffold

Bone is a three-dimensional non-homogeneous structure with complex characteristics ranging from macroscopic to the nanoscale. A sound design approach is needed that combines structural stiffness with fluid permeability so that the scaffold is both permeable enough to transport nutrients and rigid enough to resist physical loading. Completely solid metals are not compatible because they are impermeable, and the Young’s modulus of solid metals is much higher than the bone modulus of the human body. Because of the mismatch in stiffness, a stress shielding phenomenon will occur, resulting in scaffold failure. Recently, porous structures have been introduced into orthopedic surgery to replace damaged bone tissues. If the structure of porous metals can be digitally designed and fabricated with advanced manufacturing techniques, they can be designed to replicate the properties of bones [17].

It is almost impossible to analyze quantitatively the properties of conventional porous scaffolds because they are composed of a large number of randomly shaped pores. In order to obtain a simplified model, researchers assume that the scaffold is composed of periodically repeating unit cells. CAD, image-based design, and implied surfaces are the conventional design strategies for typical cyclical scaffolds. The CAD-based approach is the most commonly used method in scaffold design, mainly using various CAD tools such as Unigraphics NX (UG),Computer Aided Tri-Dimensional Interface Application (CATIA), and Pro/ENGINEER (Pro/E) [18,19]. To simplify the design process based on CAD, several specialized design software packages have been developed containing libraries of widely used construction units. Based on the scaffolding libraries, the Computer Aided System for Organizing Scaffolds (CASTS) was developed. The aim was to achieve effective automation of the entire design process for the desired topology [20,21]. Shape functions are used to construct porous supports with implicit functional surfaces or irregular polygon models in mathematical modeling, breaking through the geometric limitations of conventional porous units. Although scaffolds with ideal stiffnesses and permeability can be obtained using these methods, these methods require extensive experiments to achieve the desired performance. Moreover, the final results obtained may not be optimal [22].

### 2.1. Solid Isotropic Material with Penalization (SIMP) Method

**Figure 1:**
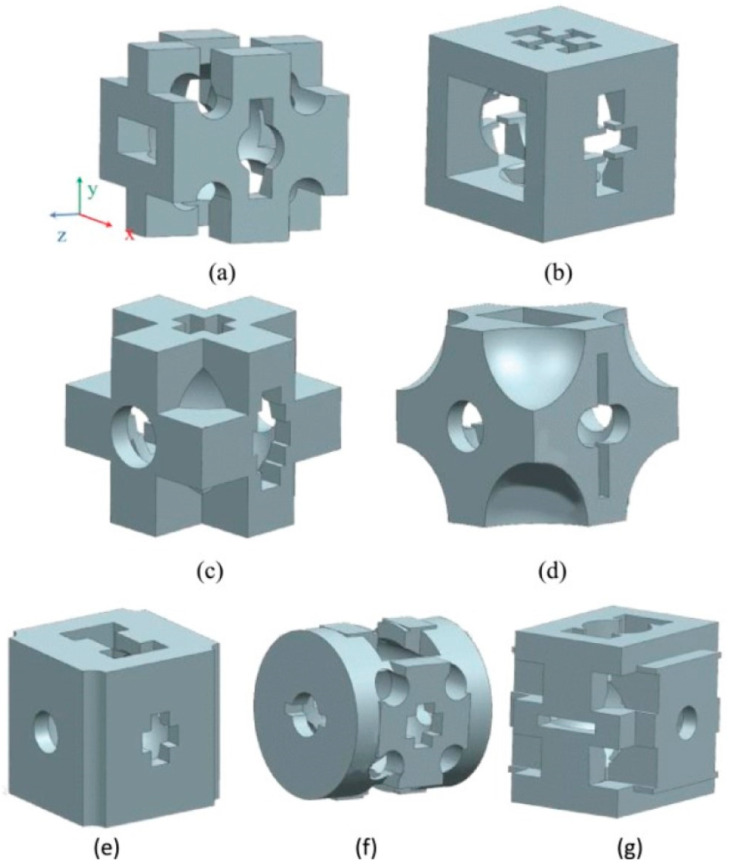
Cell model after optimization at different positions: (**a**) M15; (**b**) M16; (**c**) M17; (**d**) M18; (**e**) M19; (**f**) M20; and (**g**) M21 (numbers represent different positions) (Adapted with permission from Liu et al., 2021) [24].

Currently, there are numerous types of topology optimization methods. In the case of the SIMP method, it is a material interpolation model that allows for the existence of intermediate relative densities (between 0 and 1), penalizes the material density, and filters low-density cells to obtain accurate topological results. This method, which is of great power, can design complex structures with multiscale features [23]. A cell optimized by the SIMP method is shown in Figure 1.

The SIMP method was used to design the porous structure using the customized morphology and mechanical properties of trabecular bone to design a three-dimensional structure with gradient porosity similar to the pore structure [24]. In order to obtain the desired porosity and elastic properties, a homogenization-based algorithm was used to design a three-dimensional bone scaffold [25]. The authors also demonstrated that the method can produce a porous structure that matches the anisotropic stiffness of human trabecular bone using recognized biomaterials. Porous structures with maximum permeability have been designed by this method [26]. Researchers also optimally designed multifunctional porous material microstructures for two competing properties, namely, stiffness and fluid permeability [27]. The topological optimization technique was used to minimize the difference between the effective elastic tensor of the optimized scaffold and the elastic tensor of natural bone. By comparison, it was concluded that bone remodeling was optimal when the elastic tensor of the bone scaffold was slightly higher than that of natural bone [28]. Despite the advantages of the SIMP method, the resulting optimized structures generally suffer from numerical instabilities such as tessellation, grid dependence, and grayscale cells. Moreover, there is no way for pore connectivity to be ensured by the material model in the microstructure design, and additional non-physical constraints are needed during the optimizations [29].

### 2.2. The Voronoi Method

Voronoi tessellation method (VTM) is a way of modeling irregular open-hole structures that can be used to delineate spatial regions [30].

In previous studies, a method that allows for the design of interconnected porous lattices was proposed, which can mimic specific tissue characteristics to achieve bone regeneration scaffolds [31]. The method combined the anatomical shape of the defect by controlling the porosity and pore size of the scaffold. The advantage of this method is to provide geometrical heterogeneity, thus resulting in a very biomimetic shape.

**Figure 2:**
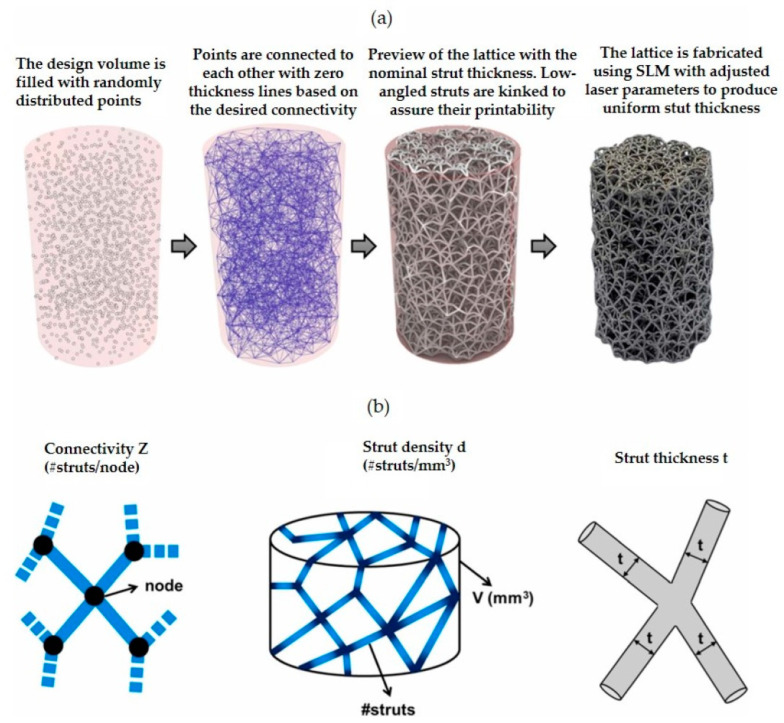
(**a**) Design and manufacturing workflow of stochastic lattice structures. The specimen displayed was designed with connectivity *Z* = 12, strut density *d* = 5, and strut thickness *t* = 230 µm. (**b**) Sketches of the design parameters (Adapted with permission from Kechagias et al., 2022) [34].

A parametric design method for lattice porous structures was proposed based on the design characteristics of Voronoi structures [32]. Deviations in model porosity and surface area are ensured because the uniform distribution of seed points has a high stability in the lattice cells. By this method, not only lattice structures with uniformly fractionated or graded distribution of porosity can be generated, but also lattice structures with customized porosity according to each cell can be generated. A VTM-based structural design method was proposed in order that the dominant elastic modulus of the scaffold could be controlled, and the stress shielding between the scaffold and the bone could be reduced [33]. A gradient scaffold suitable for the natural bone modulus can be obtained by this method, which also improves the stress shielding. It was found that the stochastic structure can be defined independently by nodal connectivity *Z*, strut density *d*, and strut thickness *t* during the design phase. The relative density, stiffness, and ultimate strength of the structure can also be predicted based on the parameters [34]. The design flow of the structure is shown in Figure 2. The advantage of the stochastic structure is that the single integrated model, presented in this study, can define the structure to achieve a broad range of design requirements, even as a gradient of properties within the same component.

The relationship between porosity or apparent elastic modulus and compressive strength of irregular porous structures cannot be simply generalized, as an increase in one parameter leads to a decrease in the other parameter. A more complex relationship may exist based on the complex irregular porous structure of VTM and needs to be further investigated [35].

### 2.3. The Machine Learning Method

Machine learning (ML) has proven to be a valuable method for research in various fields. It is used to discern patterns from complex data sets and is a branch of artificial intelligence. ML algorithms are used in areas such as image and speech recognition, spam detection, and drug discovery [36]. The properties of materials or structures can already be predicted by machine learning models, and new materials with the desired properties can also be designed.

**Figure 3:**
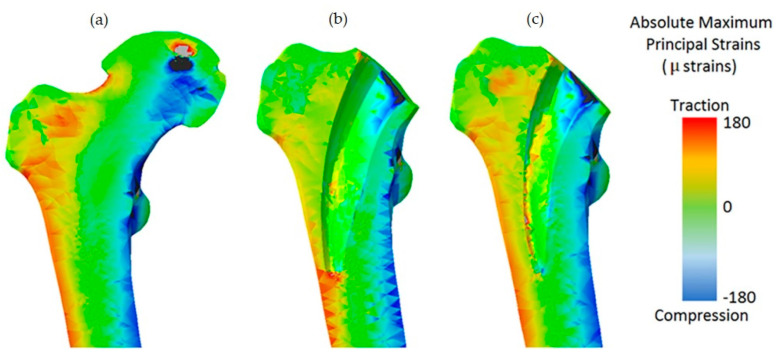
(**a**) Comparison of the absolute maximum principal strain distribution between the intact bone model, (**b**) the one implanted with the original stem, and (**c**) the new design (Adapted with permission from Cilla et al., 2017) [37].

Machine learning techniques (MLTs) were combined with parametric finite element analysis (FEA) to further optimize the geometry of the short-stemmed hip scaffold to reduce proximal femoral stress shielding [37]. The minimization algorithm was used to obtain the optimal geometry, which allows unseen values of selected parameters of the hip brace geometry to be explored. The combination of FEA, MLT, and search pattern optimization algorithms can significantly reduce the computational cost [37]. The optimized scaffold design is shown in Figure 3. An efficient method for the design optimization of scaffolds in biological tissues was proposed, which reduced computational time [38]. The optimization problem for the design of the geometry of titanium scaffolds was formulated by introducing a probabilistic model of mesoscale cortical bone. With this advanced algorithm, this very difficult constrained nonconvex optimization problem can be solved in the presence of uncertainty in biomechanics. A new method for designing layered materials using machine learning was proposed [39]. A database of hundreds of thousands of structures from FEA was used for training, along with a self-learning algorithm for discovering high-performance materials in which inferior designs are eliminated to obtain superior candidates. It further demonstrated that coarse graining can be replaced by machine learning, which means that materials can be analyzed and designed without the use of complete microstructural data. New material designs can be discovered and fabricated by this new paradigm for intelligent additive manufacturing with several orders of magnitude improvement in computational efficiency over conventional methods. A Generative Adversarial Network (GAN) model was proposed to learn the Inverse Homogenization (IH) mapping from attributes to cell shapes that can be used to optimize functionally graded cell structures [40]. Machine learning algorithms were used to predict the most suitable polymer/blend for cartilage replacement, using as input a range of tensile modulus, elongation at break, and tensile strength of natural cartilage [41].

However, as the number of parameters increases, the time required for MLT increases exponentially. As a data-driven model, IH-GAN cannot generate the shape and properties of cells outside of a given training data distribution. This may limit the performance of the optimized cell structure [40].

### 2.4. The Genetic Algorithm (GA)

**Figure 4:**
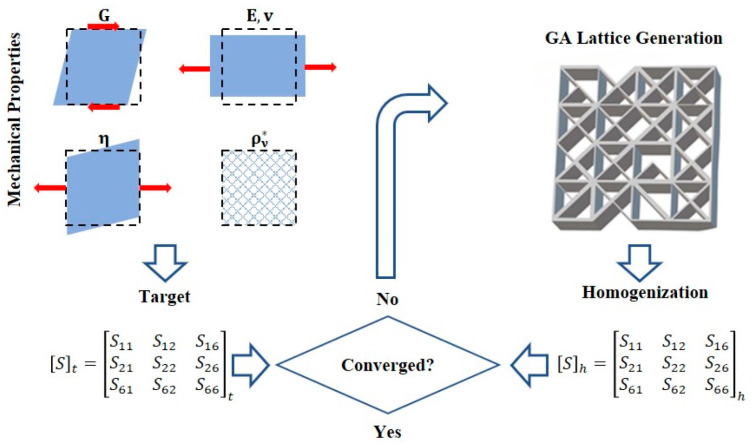
Inverse homogenization iterative process: The combined genetic algorithm and homogenization based scheme identifies inner material architectures that can optimally meet different target macroscale material properties $\left(G,E,\nu ,\eta \right)$, as encapsulated in the compliance homogenized tensor $S_{h}$, up to convergence with the target compliance tensor $S_{t}$ (Adapted with permission from Dos Reis et al., 2022) [46].

Genetic algorithms are widely used in many structural optimization designs due to their high efficiency [42]. Therefore, in many current research works, genetic algorithms are used to obtain the optimal bracket structures. For example, a new computer method was developed that combines FEA and GA to design the scaffold by selecting the scaffold fiber diameter and inter-fiber spacing to show the required stiffness for each degradation stage [43]. The Kriging (KRG) method was used for multi-objective optimization using the Non-Dominated Sorting Genetic Algorithm II (NSGA-II) to obtain the optimal design of hierarchical three-dimensional porous (H3DP) structure with the best crush resistance [44]. Based on the structure of the multi-constrained knapsack problem modeled as an ellipsoid, the inverse model of the porous structure was solved by a hybrid genetic algorithm. The bionic bone scaffold generated had good bioactivity, better mechanical properties, and a uniform degradation rate [45]. A numerical method for metamaterial reverse engineering was proposed that combines an asymptotic homogenization scheme with a genetic algorithm that can determine the optimal internal material pattern using the complete set of parameters contained in the target compliance tensor [46]. The inverse homogenization iterative process is shown in Figure 4. By integrating finite element analysis and multi-objective GA, a novel multi-objective custom shape optimization scheme was developed for cementless femoral scaffolds [47]. An optimization framework was proposed for generating readily available preoperative planning solutions in a fully automated manner [48]. It was based on a genetic algorithm capable of solving multi-objective optimization problems with nonlinear constraints. A GA-based search was carried out to optimize the scaffolds by minimizing the back-propagation neural network (BPNN) predicted micromotion. The best MMGs obtained based on the GA search provided better primary stability compared to the initial design [49].

Although the genetic algorithm is a very powerful tool, the need to evaluate the constraint and fitness functions for each generation in the process of scaffold optimization by the genetic algorithm is time-consuming, especially in the calculation of scaffolds with complex structures.

### 2.5. Other Methods

A comparison of the advantages and disadvantages of the above-mentioned four methods is shown in Table 1. In addition to this, there are other ways to optimize the bone scaffold. The level set algorithm is centered on tracking phase boundaries, thus effectively describing smooth boundaries to control topological variations [50]. A more systematic and comprehensive study of topology optimization based on level sets was conducted [51]. In order to obtain materials with maximum magnetic permeability, a variable level set technique was developed for the periodic material design problem controlled by the Navier–Stokes and Maxwell’s set of equations [52]. The Bi-directional Evolutionary Structure Optimization (BESO) method allows for the simultaneous addition and removal of materials during the optimization process [53]. In the present study, only some of the optimization methods regarding bone scaffolds were reviewed, but not all of them were presented. The mechanical analysis and optimization of equations for bone and bone implants were also not mentioned.

## 3. Current Settings in the Optimization of Bone Scaffold

### 3.1. Setting of Objective Function

The overall layout structure of the scaffold should be consistent with the homologous anatomy. Different material distribution but the same mass or volume of the metallic material will result in different biomechanical properties. Most studies have two main goals for the overall layout structure of the topology optimization design. The scaffold is described as a structural support framework that provides a temporary, artificial extracellular matrix for the growth of new tissue, preferably with properties similar to those of the host bone [54,55]. Therefore, the objective function is usually formulated as the difference between the target stiffness and the corresponding entry of the bone scaffold stiffness value. The stress shielding caused by the mismatch between the mechanical properties of the bone and the scaffold has been reduced. In orthopedic scaffolds, the strength of porous scaffolds should be close to that of human bone in terms of mechanical properties to provide strong mechanical support for damaged bone. Various mechanical parameters of bone scaffolds are investigated according to the injured bone tissue and its loading conditions. Among other things, since bone growth is regulated by the mechanical environment, the Young’s modulus of the scaffolds has a crucial role to play in enhancing bone formation [56,57]. The Young’s modulus of natural bone tissue is usually lower than that of metallic alloys. For example, Ti-6Al-4V has a modulus of elasticity of about 110 GPa compared to 0.02 to 2 GPa for bone trabeculae [58]. This Young’s modulus mismatch may lead to stress shielding, osteoporosis, and fracture [59,60]. Therefore, the mechanical behavior of the open porous stent must be adjusted to reduce the risk of loosening of the scaffold through stress shielding. Liu et al. [24] provide a new design method that can simulate the anisotropic microstructure of bone tissue.

The second goal is usually to reduce the weight of the bone scaffold. Solid metal devices may be too heavy to maintain body balance. Many studies have demonstrated that the reduction in mass or volume allows for more scaffold material and more room for tissue to grow inward. Specially tailored structures are more conducive to bone surface adaptation and weight reduction than modular scaffolds [61,62]. Within the same design domain, an optimized design using less material can achieve a better mechanical structure while also reducing the weight of the bone scaffold and making it more acceptable to the patient. A three-dimensional tetrahedral titanium scaffold was used to reconstruct a mandibular defect, achieving a minimal weight design [63].

### 3.2. Setting of Design Variables

After the objective function is selected, the desired objective can be easily reached by adjusting different design variables (Table 2). In a certain design domain, many points are randomly distributed. These points are called seed points. The seed points are used as the core to generate many small irregular polyhedral structures. The common edges of the polyhedral are used to generate the beam structures. Many designers expand many other design variables based on the number of seed points and the radius of the bus bar of the beam structure. The number of seeds (*n*) contained in the scaffold envelope box and the scale factors S_f_ and S_v_ are chosen as design variables [31]. By maintaining the same number of seeds and varying the scaling coefficient from 0 to 1, the scaffold sample can be derived and the percentage porosity measured. The structural parameters of regular lattice structures as well as the structural parameters of uniform lattice structures and the porosity function relationship are proposed by taking the number of seed points *n* and the beam radius *r* in the structural parameter lattice cell as design parameters [32]. The random number *R*, the seed number *N* and the scaling factor *F* are chosen as design variables [33]. Kechagias et al. [34] chose the strut density as a design parameter, and since it can ultimately be related to the density *ρ* of the structure, using it to evaluate the number of nodes required makes the design more reasonable. The performance of the irregular porous bracket can be adjusted by the three design parameters (strut diameter *D*, unit distance *d*, and irregularity *i*) [35].

The geometric parameters of the scaffold are often selected as design variables to optimize its mechanical properties. Machine learning and genetic algorithms used to optimize bone scaffolds usually select geometric dimensions related to the scaffold geometry as design variables [37,38,47]. Therefore, the number of design variables is relatively small. However, most topology optimization methods use pixels or nodes, which involve large and time-consuming design variables [64,65]. The design domain is first discretized into pixels or cells with a reasonable resolution, and then the topology of the structure is represented by finding the presence or absence of material on each cell based on an optimization algorithm. Therefore the computational effort to find the most structure designed by pixel-based design variables is relatively high, especially when considering three-dimensional problems [66]. The relationship between computational efficiency and structural design will need to be addressed in the future [67].

### 3.3. Setting of Constraints

#### 3.3.1. Pore Size

One of the important features of 3D-printed scaffolds for bone regeneration is the pore size [69]. The pore size of porous structures directly affects the transport and exchange of nutrients and blood, cell adhesion and proliferation, cell interaction, and even bone formation, so whether the pore size is suitable is an important index for judging porous structures. However, in the existing studies, due to the different experimental subjects, experimental conditions and experimental operations, researchers have different or even contradictory statements about the optimal pore size, and there is no uniform standard for the time being. Studies have shown that only large pore size (300–800 μm) can ensure the successful completion of the bone growth process in implants or scaffolds [70]. Large pore size is conducive to the transfer of nutrients and the discharge of metabolic waste, and cells can obtain more nutrients, which is conducive to cell growth. Ran et al. [71] fabricated simple porous Ti6Al4V scaffolds with pore diameters of 400 μm, 600 μm, and 800 μm, and they found that the 600 μm scaffold had good stability and facilitated inward bone growth. However, larger pore size is not better. Too large a pore size may reduce the interaction of cells with the porous structure. Fukuda et al. [72] found that at pore sizes greater than 1000 μm, the bio response level of porous scaffolds decreased, and osseointegration was significantly weakened, with 500 μm showing the best bone ingrowth effect. Feng et al. [73] found experimentally that scaffolds with pore sizes greater than 400 μm were more inclined to form mineralized bone directly. Therefore, the pore size range of porous structures should be 500–1000 μm.

#### 3.3.2. Porosity

**Figure 5:**
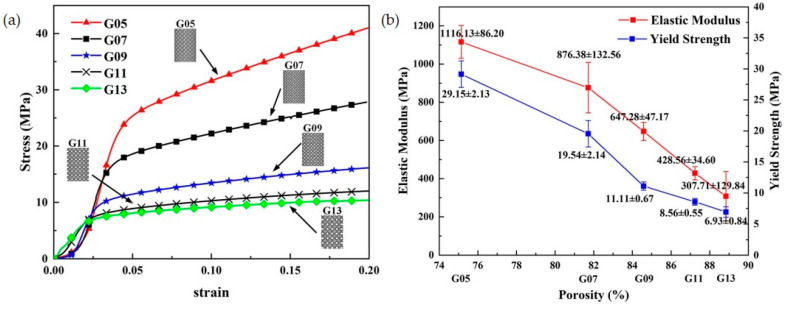
(**a**) Nominal stress–strain curves of G05–G13 structures (G represents gyroid). (**b**) Drop curve between porosities and measured elastic moduli and yield strengths of as-built samples (Adapted with permission from Ma et al., 2020) [75].

In the design domain, the pore volume as a percentage of the total volume is the porosity. Having a scaffold with porosity allows cells to migrate smoothly and can improve the surface area for interaction with surrounding tissues. Theoretically, the higher the porosity, the more space is available for cell growth and migration, and the more cells are delivered for tissue repair, the more it can help new bone growth. TPMS structures with 80–95% porosity and moduli in the range of 0.12–1.25 GPa, which converge to the modulus of trabecular bone, were fabricated by the SLM technique. The effects of porosity and pore size on mechanical properties and permeability were investigated by experimental and simulation methods [74]. Researchers found that permeability increased with increasing porosity and pore diameter, and that the scaffold had the highest number of cells when the porosity was 88.8%; however, the mechanical properties decreased [75], as shown in Figure 5. However, some studies demonstrated that high porosity allows better cell migration and growth, but it is difficult to maintain the optimal mechanical strength [76]. Thus, we need to make a compromise between mechanical strength and porosity as well as pore size in designing bone scaffold engineering. The connected porosity of cancellous bone is between 60% and 80%, so the porosity selection range should be between 60% and 80% in porous structures.

#### 3.3.3. Pore Shapes

**Figure 6:**
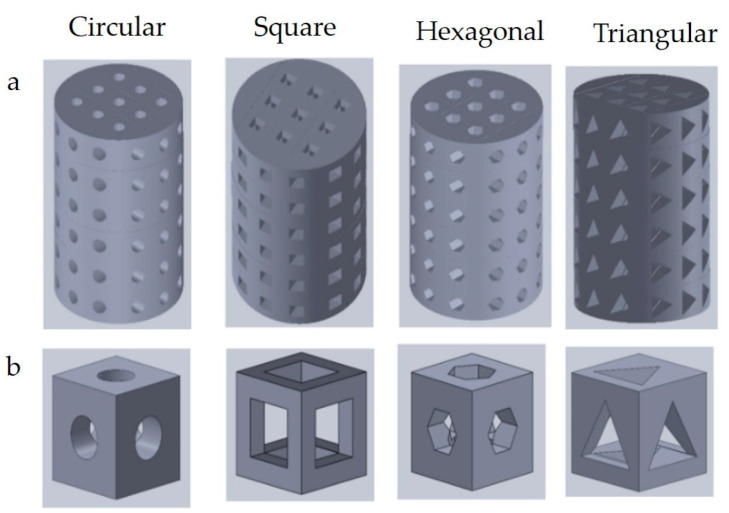
(**a**) Scaffold with different pore shapes and (**b**) unit cells (Adapted with permission from Jahir-Hussain et al., 2021) [79].

Different pore shapes have different effects on the mechanical and biological properties of scaffolds. The 3D printed scaffolds with different pore geometries are shown in Figure 6. Two different 3D printing materials were studied to find the effects of pore geometry on cell enrichment and differentiation. It was found that scaffolds made with cubic pores increased the gene expression of cells more than those with cylindrical pores [77]. The cyclic stress–strain responses of scaffolds with triangular and circular pores were compared (Table 3) [78]. The scaffold with circular pores was found to exhibit better fatigue resistance in terms of cyclic damage. The use of different pore shapes as constraints can yield different skeletal inward growth properties.

#### 3.3.4. Additive Manufacturing Constraints

Although additive manufacturing techniques can theoretically allow for structures of any shape, the quality of the structure is highly dependent on the design parameters [73].

- Overhead Structural Constraints

Additive manufacturing is a “bottom-up” material layer-by-layer manufacturing method, which makes it possible to process overhanging structures with only powder as support (SLM, EBM) or no support at all in fused deposition modeling (FDM) [80,81,82]. Practical experience shows that when the angle between the lower surface of the overhanging structure and the printing platform is less than the critical angle (usually 40–50°), the overhanging structure is prone to collapse or warping and deformation due to insufficient support underneath, resulting in processing failure. Therefore, there are often strict overhang constraints in additive manufacturing, and sufficient support must be added underneath when processing overhanging structures that do not meet the angle requirement. The added support structure is shaped during machining and needs to be removed during post-processing, which means that additional material and machining time is inevitably wasted. Therefore, to reduce the number of supports in the manufacturing process, several authors have proposed different optimization methods for the support structure.

Using a large number of SLM printing experiments and statistical analyses of variance experimental results, the design rules for optimizing the support structure of the commonly used metal printing materials 316L stainless steel and Ti6Al4V in SLM were summarized [83]. A shape function-based optimization algorithm for the forming orientation of additively manufactured parts was developed, which determines the optimal forming orientation of a part using the minimum number of overhanging structures to be supported as the optimization criterion [84]. The strength of the support structure can be adjusted by changing the porosity of the support structure through the shape function. The strength of the support structure can be adjusted by changing the porosity of the support structure through the shape function. The number of supports of the optimized part can be reduced by 42% using this method.

- The Accuracy Constraints

Parts can be classified as consisting of basic structural features, such as planes, surfaces, columns, spheres, holes, angles, thin walls, gaps, etc. These structural features carry the functional characteristics of the part, and their forming quality becomes the key to determining the performance of the part. Since additive manufacturing technology is not all-powerful, its ability to form structural features is limited by a variety of factors, such as the negative impact of processing equipment accuracy, and process parameter errors on forming accuracy are common in additive manufacturing. Moreover, these factors can cause differences in additive manufacturing molding accuracy due to differences in materials and printing processes. The problem of molding accuracy constraints has been a factor limiting the development of additive manufacturing technology [85]. For example, the forming accuracy of FDM is limited by the diameter of the nozzle (generally 0.2–0.6 mm); SLM affects the surface accuracy due to the presence of a certain thickness of the powder layer (30–50 μm), which forms a “stepped surface” on the inclined surface of the part; the large laser spot diameter (70–100 μm) of SLM makes it difficult to shape fine characteristic structures such as sharp corners, and this problem is more prominent in Laser Cladding Forming (LCF) technology [86,87].

- Connectivity Constraints

Additive manufacturing requires the removal of residual uncured resin, unmelted powder, and auxiliary support depending on the process. For structures with internal closed cavities, the residual material cannot be removed, so secondary modifications or partitioning of the structure is often required, which greatly increases the manufacturing process’s difficulty and cost [88]. In order to ensure that the excess material inside the cavity structure can be removed smoothly during post-processing, the structure needs to be connected and cannot contain a closed cavity structure inside. A method to create a virtual temperature field to describe the structural connectivity was proposed, i.e., assigning high thermal conductivity and adiabatic material properties to the cavity structure and solid structure, respectively. When a closed cavity is present in the structure, a rapid increase in local temperature is induced. The connectivity control of the structure is achieved by constraining the overall maximum temperature [89,90].

## 4. Challenges in the Optimization of Bone Scaffolds

### 4.1. Design of Bionic Bone Scaffolds

The real bone trabecular structure is a complex and irregular porous structure, which can only be designed using the irregular porous structure design method and needs to achieve a close approximation to the real bone trabecular in terms of morphology, characteristic parameters, mechanical properties, and biological properties to achieve replacement, which is eventually used to treat bone defects, comminuted fractures, and other orthopedic diseases. The bionic structure of porous scaffolds is characterized by SEM and micro-CT [91]. Random porous structures can lead to improved bone endogenous growth processes because they resemble trabecular bone in appearance [92]. Non-homogeneous porous structures based on natural irregular patterns are constructed, and the results show that such structures can promote bone regeneration in vivo and in vitro [93]. Most irregular porous scaffolds have been developed by mimicking natural skeletal features through image-based inverse modeling approaches, mathematical modeling approaches, or a combination of them [94,95]. Unfortunately, the effects of irregularity and its evolution have been mostly ignored in previous studies.

### 4.2. Validation of Numerical Simulations

Optimization is based on digital models simulating the real situation, which cannot summarize all the information of the sample, and there must be many simplifications and assumptions, such as most of the models do not introduce muscles and ligaments, or they replace them with simplifications, thus affecting the accuracy of the results [96]. The optimization process often requires reasonable simplifications and assumptions to improve computational efficiency. It is assumed that the material of the optimization object is homogeneous, isotropic, and linearly elastic, such that muscle and ligament are often substituted, so the calculation results will be somewhat different from the actual situation. Therefore, the computational results derived using the finite element model also need to be analyzed against biomechanical experiments and compared with clinical data, thus providing a cross-fertilization effect. The topological optimization of nonlinear materials needs to be further explored.

### 4.3. Multi-Objective Optimization

Considering multi-material and multi-scale optimization models in bone scaffold optimization, using different optimization methods in performing model optimization, and joint optimization of bone scaffolds based on macroscopic and microscopic scales are essential for the lightweight and mechanical properties of bone scaffolds [47]. With the development of medical and engineering integration, the improved design of in vivo scaffolds combining multi-objective optimization methods should be vigorously developed [97]. Multi-objective optimization is an important part of different optimization methods toward engineering design practice and faces difficult problems such as load pathology, sensitivity construction, and multi-objective unification. The existing design guidelines have difficulty describing the typical characteristics of the mechanical response of the bone support structure throughout the degradation time course, and the approximate multi-objective optimization carried out based on them does not guarantee the most desirable results as the complexity of the structure is enhanced [98]. Therefore, there is a need to define more applicable optimization criteria based on mechanical and biological characteristics to effectively guide the multi-objective optimization of complex bone scaffold structures.

### 4.4. Integrated Design and Manufacturing Performance

Considering the capability of current additive manufacturing technology, the optimized bone scaffold structure may have a local structure that is not suitable for additive manufacturing, the initial structure needs to be modified for manufacturability, and the modified structure may have the problem of destroying the optimized optimal configuration and affecting the optimal design [99]. Therefore, research on multi-scale, multi-faceted lightweight structures and multi-material gradient layout optimization design methods is needed for additively manufactured bone scaffolds.

### 4.5. Incorporating Degradable Behavior into the Optimization Process

It is worth mentioning that most optimization methods do not consider the degradation of the scaffold, as it usually occurs at a later stage of bone remodeling [100]. What needs to be considered in the future is to incorporate the process of scaffold degradation into the optimization, which could allow us to understand how to control tissue growth as well as the rate of bone scaffold degradation to achieve optimal remodeling performance. An ideal scenario would be for the optimization algorithm of the scaffold structure to be composed of three modules, namely, the new tissue forming module, the bone scaffold degradation module, and the optimization module for the scaffold.

## 5. Conclusions and Future Prospects

In conclusion, various numerical methods for designing bone scaffolds are presented in the present study. In the past few years, various methods have been developed to design bone scaffolds with complex internal structures. In the design setup of bone scaffolds, special attention should be paid to the pore shape, the pore size, etc. The current challenge is to design bionic bone scaffolds with better performance and longevity. Optimization of bone scaffolds is a dynamic and promising research area. The future developments in the design of bone scaffolds are as follows:

(1) Currently, the research and application of digital twin technology are still in their infancy. There are various possibilities for its combined application with additive manufacturing and topology optimization, such as modeling techniques using digital twins and the application of additive manufacturing technology. The integration of these new technologies with different optimization methods will lead to breakthroughs in the field of optimization of the bone scaffold.
(2) The manufacturing and clinical application of personalized implants are still in their early stages, owing to the process of developing the medical 3D printing materials required for implant manufacturing. The current bone implants are mostly based on metal materials. More high-performance materials that conform to the elastic modulus of human bones need to be investigated and developed.
(3) Optimization techniques based on finite element analysis have been used to simulate the mechanical interaction between bones and implants outside the human body. However, there is a lack of evidence to support in vivo studies. Therefore, biomechanical research work in the human body is needed in the future to make the optimization method more clinically relevant.

The optimization of the bone scaffold is a dynamic and prospective research area. In the future, many new techniques and methods will be emerged to solve the current challenges. For example, in terms of the method, one of the most prospective design methods might be the machine learning-based method.

## Figures and Tables

**Table 1:** Comparison of different optimization methods.

	Advantages	Disadvantages	References
The Voronoi method	Excellent structure, good distribution of voids	Complex structure, complex relationship between parameters	[31,32,33,35]
The Machine learning method	Low calculation cost	High demand for training data	[38,40,41]
Genetic algorithm	Good scalability, simple process, fast convergence	Complex programming, high experience requirement for parameter selection, slow speed	[43,44,47,48]
The SIMP method	Good mechanical properties	High calculation cost, complex programming, slow calculation speed	[24,25,26,27]

**Table 2:** Comparison of design variables.

Design Variables (Examples)	Advantages	References
Number of seeds (*n*), polyhedrons faces scale factor S_f_, polyhedrons volume scale factor S_v_	Provide geometrical heterogeneity Really biomimetic shape	[31]
Total stem length L, radius of the lateral side cross section R_1_, radius of the medial side cross section R_2_, internal length D	Low calculation cost	[37]
Finite element mesh or node	Widely used Simple and reliable	[17,68]
Geometric parameters of the transverse section of the stem	Multi-objective optimization A lot of options	[47]

**Table 3:** Impact of design constraints on bone scaffolds.

Constraints	Possible Issues if Higher Than the Recommended Value		Possible Issues if Below the Recommended Value		Recommended Value
Pore size	Low intensity, weak osseointegration ability		Slow transfer of nutrients, slow excretion of metabolic waste, not conducive to cell growth		500–1000 μm
Porosity	Reduced mechanical properties, high manufacturing difficulty		Unfavorable for new bone growth		60–80%
Pore shapes					Round
			

## Data Availability

The data presented in this study are available upon request from the corresponding author.
